# An Update on the Superior Cerebellar Artery Origin Type

**DOI:** 10.3390/medicina59122164

**Published:** 2023-12-13

**Authors:** Ana-Maria Davidoiu, Mihai Lazăr, Alexandra Diana Vrapciu, Petrinel Mugurel Rădoi, Corneliu Toader, Mugurel Constantin Rusu

**Affiliations:** 1 Doctoral School, Faculty of Medicine, “Victor Babeş” University of Medicine and Pharmacy, RO-300041 Timișoara, Romania; ana-maria.davidoiu@umft.ro; 2 Department 2, Division of Physiopathology II, Faculty of Medicine, “Carol Davila” University of Medicine and Pharmacy, RO-020021 Bucharest, Romania; mihai.lazar@umfcd.ro; 3 Department 1, Division of Anatomy, Faculty of Dentistry, “Carol Davila” University of Medicine and Pharmacy, RO-020021 Bucharest, Romania; 4 Department 6–Clinical Neurosciences, Division of Neurosurgery, Faculty of Medicine, “Carol Davila” University of Medicine and Pharmacy, RO-020021 Bucharest, Romania; petrinel.radoi@umfcd.ro (P.M.R.); corneliu.toader@umfcd.ro (C.T.); 5 Clinic of Neurosurgery, “Dr. Bagdasar-Arseni” Emergency Clinical Hospital, RO-041915 Bucharest, Romania

**Keywords:** basilar artery, cerebellar artery, posterior cerebral artery, circle of Willis, duplication, fenestration

## Abstract

*Background and Objectives:* The microanatomy of the superior cerebellar artery (SCA) is clinically significant. We, thus, aimed at patterning unilateral and bilateral possibilities of SCA origin. *Materials and Methods:* In total, 205 archived records of computed tomography and magnetic resonance angiograms were used. There were defined types of SCA origin from the basilar artery (BA): “0”—absent SCA, “1”—preterminal, “2”—collateral SCA, with SCA appearing as a terminal branch of BA, and “3”—SCA from the posterior cerebral artery (PCA) of the cerebral type. Fenestrations and duplications of SCA were recorded. Bilateral combinations of types were recorded as follows: A (1 + 0), B (1 + 1), C (1 + 2), D (1 + 3), E (1 + duplicated SCA), F (2 + 2), G (2 + 3), H (3 + 3), I (3 + duplicated SCA), J (1 + fenestrated SCA). *Results:* Type 0 SCAs were found in 0.25%, type 1 in 71.29%, type 2 in 19.06%, and type 3 in 9.41%. Absent and fenestrated SCAs were each found in a single case. The most frequent combinations were B (58.05%), C (13.17%) and F (13.17%). Bilateral symmetrical types occurred in 70.7% of cases. Fetal types of PCA and the artery of Percheron modified the BA ends. Combinations of C, F, and G changed the BA ends or tips; thus, different subtypes resulted in five BA bifurcation patterns, including five BA trifurcations and one BA quadrifurcation. BA trifurcation was also found in cases with duplicated SCAs. *Conclusions:* The SCA has various anatomical possibilities of origin and bilateral combinations that are not presented in anatomical lectures. Details on the specific end of the BA should be gathered on a case-by-case basis.

## 1. Introduction

The superior cerebellar arteries (SCAs) are the final collateral branches of the basilar artery (BA) [1,2,3]. Then, the BA typically divides into the posterior cerebral arteries (PCAs). The SCAs are the most rostral pair of infratentorial vessels [4]. Few studies have investigated anatomical variations in the cerebellar arteries, and the prevalence of such variations has not been reported [5]. The SCA may be distorted by avascular masses in the posterior fossa or altered pathologically by aneurysms, arteriovenous malformations, atheromatous disease, or vascular neoplasms [6].

The SCA supplies the tectum, cerebral peduncles, the superior surface of the cerebellar hemispheres, the superior vermis, and the cerebellar peduncles [3,7]. SCA’s presence, origin, and course are considered constant [8]. In 90% of cases, the SCA arises as a single trunk [9]. In 82% of cases, the proximal segment of the SCA sends off perforator branches, which are of paramount surgical importance when treating lesions located at the tentorial notch, posterior incisural space, pineal region, parasplenial area, cerebellopontine angle, brainstem, and cerebellum [9]. The presence of perforating branches of the SCA might have been underestimated previously for the following several reasons: the earliest anatomical studies were performed postmortem without the benefit of tracers, and, on the other hand, the small perforators are poorly traced with latex or dye [9]. These issues could account for the presence of illustrations only concerning these small vessels in previous publications and the absence of photographs [9].

Different variational possibilities of the SCA have been described in the literature: duplication, triplication, early bifurcation, fenestration, flattening, hypoplasia, origin from the PCA, and the highly tortuous SCA pathway [10,11,12,13]. The early bifurcation of the SCA has been defined as that division located in the proximal portion of the anterior mesencephalic segment of the SCA [5]. The proximal segment of the SCA is approached when treating vascular pathologies such as aneurysms of the SCA or BA and arteriovenous malformations of the posterior cranial fossa [9]. The bilateral asymmetry of the SCA origin is possible [9]. The basilar tip morphology is also modified by anatomical variations in the PCA, such as a fetal type of PCA (fPCA) that is sent off by the internal carotid artery or the artery of Percheron (aP), the thalamoperforating branch of the PCA that leaves it, when present, at a variable distance from the BA tip [14,15]. The morphology of the BA tip could affect the efficacy of mechanical thrombectomy [16]. Basilar tip aneurysms are less common in cases with fPCAs [17].

Knowledge of the origin variants of SCA is essential for neuroradiologists and neurosurgeons in improving the understanding of vascular malformations and SCA syndrome, addressing BA termination lesions, and explaining trigeminal neuralgia and BA termination variants to avoid ligation or the inadvertent severing of arteries during posterior fossa surgical approaches for aneurysms, arteriovenous malformations, tumours, epilepsy operations, temporal lobectomies, and posterior cerebral revascularisation [18]. Variants of origin of SCA are significant during diagnostic and interventional neuroradiology to avoid the incorrect diagnosis, to explain an unusual picture of posterior circulatory shock, or the possible implications of surgical and interventional procedures; different anatomical patterns may influence the occurrence of aneurysms, atherosclerosis, and posterior circulatory shock [18]. The basilar SCA aneurysm arises at the crotch of the origin of the SCA, but the neck soon takes in all of the narrow lateral portion of the BA between the SCA and the PCA [19]. We, therefore, aimed to study the anatomical and imaging possibilities of the variable origin of the SCA.

## 2. Materials and Methods

For superior cerebellar artery investigations, we used an overall batch of 205 archived records of computed tomography (CT) angiograms (190) and magnetic resonance imaging (MRI) angiograms (15). There were 118 scans in men and 87 scans in women. Inclusion criteria in the group were precise, including vascular details, complete scans, and the absence of intracranial/intracerebral pathological formations. Exclusion criteria included incomplete scans, thick sections, and detectable tumour formations.

The CT examination was performed on a 64-slice CT Somatom Definition As (Siemens), with a rotation time of 0.5 s, using a pitch of 1.2 and collimation of 1.2 mm. CareDose4D and CareKV were used to reduce the radiation dose. The examination was performed with the patient in the head-first supine position, in an inspiratory breath hold. We injected a volume of 90 mL Omnipaque (300 mg I/mL) followed by a 40 mL saline bolus with a flow of 3.5 mL/s. The scan start for the arterial phase was auto-triggered when the contrast in the pulmonary artery increased by 100 HU compared to the non-enhanced scan. The venous phase was performed with a delay of 30 s after the arterial phase. We used 5 mm and 3 mm reconstructions for primary diagnosis and no overlap and 1.5 mm reconstructions with a 0.5 mm overlap and the B31f image filter for multiplanar reconstructions, maximum intensity projection, and volume rendering images. The MR examination was performed on a 3T Magnetom Vida system (Siemens) using a Head Neck 20TCS coil. The scanning protocol used in previous studies [20] included the following sequences: t1_fl2d_sag_4 mm, t2_tse_tra_512_4 mm, t2_tse_-dark-fluid_tra_3 mm, resolve_4scan_trace_tra_p2_192_4 mm, t2_swi_tra_p2_2 mm, t1_fl2d_tra_4 mm, t2_tse_dark-fluidcor_3 mm, 3d_tof, t1_mprage_tra_p2_iso_0.9 mm. A volume of 20 mL Clariscan was injected, followed by 20 mL of saline bolus with a flow of 3 mL/s and repeated t1_mprage_tra_p2_iso_0.9 mm (with automatic subtraction of the non-enhanced sequence), followed by t1_se_tra_blood-suppr._MTC_4 mm. Multiplanar and volumetric reconstruction were performed using the t1_mprage sequence.

Anatomical variants were documented with Horos v3.3.6 software for OS X (Horos Project, Annapolis, MD, USA), on planar or curved planar sections, and via three-dimensional volumetric renderings. The principles of the Declaration of Helsinki were used to conduct the research. The Ethics Committee (affiliation #5) approved the study (approval no. 2093/1 March 2022).

**Figure 1:**
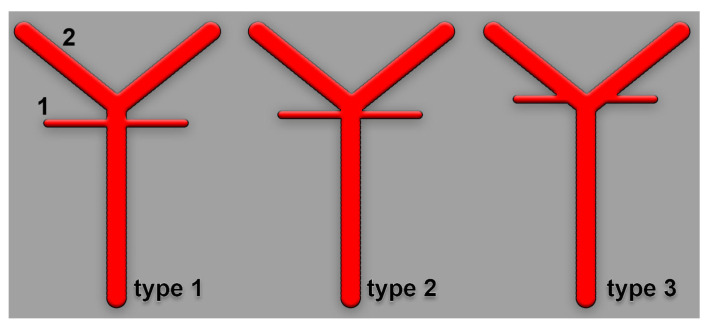
Anatomical possibilities of insertion at origin into the basilar artery of the superior cerebellar artery. Scheme. Type 1—collateral, 2—terminal, and 3—cerebral. 1. superior cerebellar artery; 2. posterior cerebral artery.

The following types were defined for the study regarding the anatomical possibilities of SCA origin: type 0, absent SCA, type 1 (typical, collateral)—with the SCA origin as a preterminal collateral branch of BA—type 2 (terminal)—SCA origin as a terminal branch of BA, either with a distinct angle between SCA and the suprajacent PCA (common origins of SCA and PCA), or with the ipsilateral absent P1 segment of PCA (BA appearing terminally trifurcated) and, implicitly, fPCA on that side—and type 3 (cerebral), with a SCA origin from the P1 segment of ipsilateral PCA (Figure 1). We also recorded SCA fenestrations and duplications. For the duplication, we noted the individual variant (types 1, 2, or 3) of each component of that duplicated SCA. Determinations were made separately for each side for the right (R) and left (L) by type (e.g., R1 indicates type 1 for right SCA). We defined the bilateral combinations of SCA types, as shown in Table 1.

## 3. Results

The prevalence of SCA types 0–3 in the overall group (410 SCAs) and by sex (236 SCAs in males, 174 SCAs in females) was determined, and due to cases with the duplication or fenestration of SCA, the sum of these prevalences resulted in <100%. In the total lot (N = 410 SCAs), type 0 occurred in 0.25%, type 1 in 71.29%, type 2 in 19.06%, and type 3 in 9.41% of cases. In males (N_M_ = 236 SCAs), type 0 was found in 0.43%, type 1 in 67.38%, type 2 in 20.6%, and type 3 in 11.59% of cases. In females (N_F_ = 174 SCAs), the aplastic type 0 of SCA was not found; type 1 occurred in 76.61%, type 2 in 16.96%, and type 3 in 6.43% of cases.

**Figure 2:**
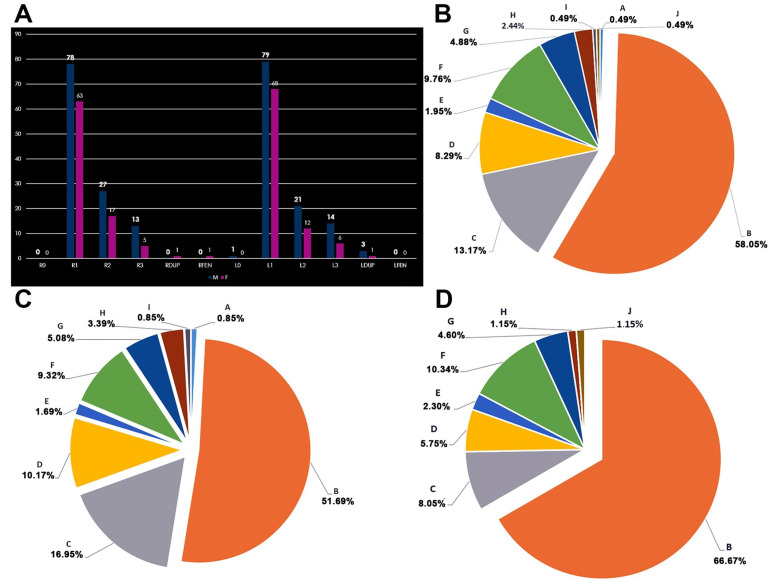
(**A**) Count of types of superior cerebellar artery (0–3), duplication (DUP), and fenestration (FEN) by sex and on either side of the median plane, right (R), and left (L). M: male; F: female. Male sublot N_M_ = 118. Female sublot N_F_ = 87. (**B**) Prevalence of anatomical A–J patterns of bilateral combinations of superior cerebellar artery origin types in the general lot. (**C**) Prevalence of A–J anatomical patterns of bilateral combinations of superior cerebellar artery origin types in the male group (N_M_ = 118). (**D**) Prevalence of A–J anatomical patterns of bilateral combinations of superior cerebellar artery origin types in the female group (N_F_ = 87).

The results obtained after documenting the SCA variables are detailed by sex and each side of the median plane in Figure 2A. In both sexes, type 1, standard, with the SCA on the left as collateral and proximal to the terminal end of the BA, prevailed. Only in one male case was the SCA absent on the left side. Only one case was identified from the female subgroup (N_F_ = 87, 1.14%) with a right SCA fenestration. Compared to the overall group (N = 205), this resulted in a prevalence of 0.48% for SCA fenestration. In the general group, five duplicated SCAs were identified (2.43%), including four on the left side (1.95%) and one on the right side (0.48%). In males (N_M_ = 118), three duplicated SCAs were identified (2.54%), all on the left side. In females (N_F_ = 87), two SCA duplications were documented, including one on the right and two on the left (2.29%).

In the overall group, type B (bilateral SCA type 1) prevailed (58.05%). This was followed by type C (SCA type 1 + SCA type 2) in 13.17% and type F (bilateral type 2 of SCA) in 9.76% (Figure 2B) of individuals.

In the male group (N_M_ = 118), bilateral type B—type 1—identified SCA combinations and prevailed in 51.69%. This was followed by type C (SCA type 1 + SCA type 2) in 16.95% and type D (SCA type 1 + SCA type 3) in 10.17% (Figure 2C) of individuals.

In the female group (N_F_ = 87), bilateral combinations of type B—type 1 SCA—were identified bilaterally and prevailed in 66.67%. This was followed by type F (SCA type 2, bilateral) in 10.34% and type C (SCA type 1 + SCA type 2) in 8.05% (Figure 2D).

Bilateral symmetric types B, F, and H were identified in the overall group in 70.7% of cases. In the male group, they were present in 64.4% of cases; in the female group, they were present in 78.16%.

**Figure 3:**
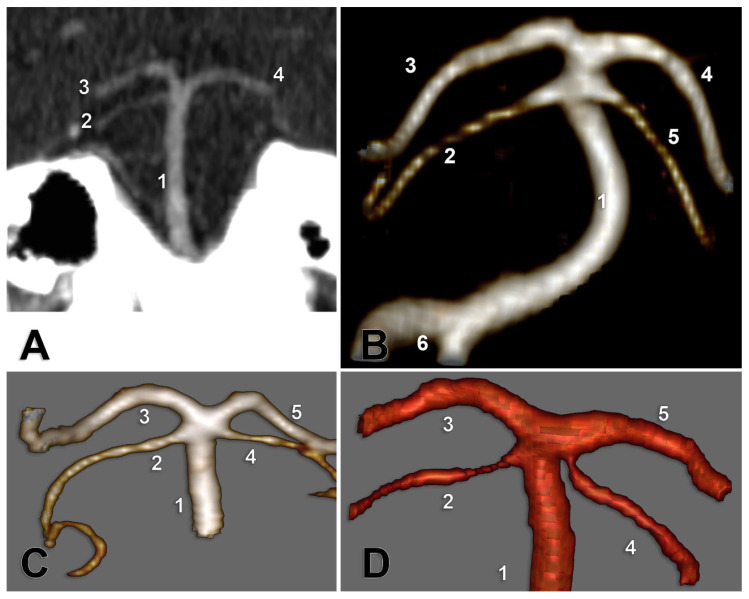
(**A**) Type A of the bilateral combination of superior cerebellar arteries origins. Coronal slice, anterior view. 1. BA; 2. right SCA; 3. right PCA; 4. left PCA. (**B**) Type B of the bilateral combination of superior cerebellar arteries origins. Posterior view of the basilar artery. CT angiogram, three-dimensional volume rendering. 1. BA; 2. left SCA; 3. left PCA; 4. right PCA; 5. right SCA; 6. vertebral arteries. (**C**) Type C of the bilateral combination of superior cerebellar arteries origins. Posterior view of the basilar artery. CT angiogram, three-dimensional volume rendering. 1. BA; 2. left SCA; 3. left PCA; 4. right SCA; 5. right PCA. (**D**) Type D of the bilateral combination of superior cerebellar arteries origins. Posterior view of the basilar artery. CT angiogram, three-dimensional volume rendering. 1. BA; 2. left SCA; 3. left PCA; 4. right SCA; 5. right PCA.

Type A of the bilateral combination indicates the unilateral absence of SCA. It was identified in only one male case (Figure 3A).

Type B of the bilateral combination (bilateral collateral types of SA) (Figure 3B) prevailed in the general lots and gender lots. Type C of the bilateral combination (Figure 3C), consisting of unilateral types 1 and 2, was the second most prevalent in the general and male lot. In type D, SCA was present on one side of type 1 (collateral), and contralateral SCA was present for type 3 (cerebral) (Figure 3D).

**Figure 4:**
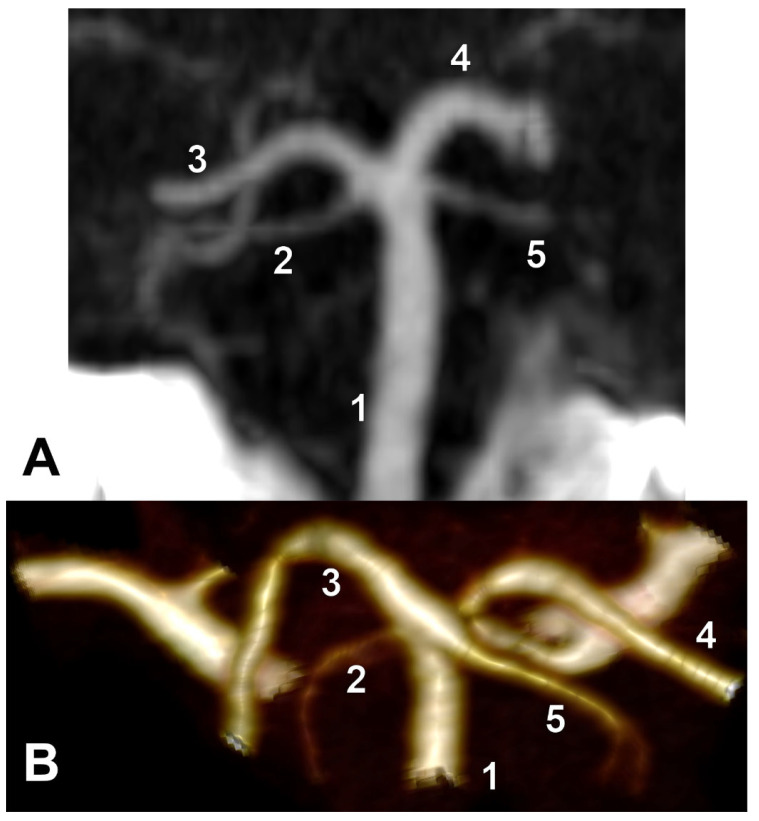
Types F of the bilateral combination of superior cerebellar arteries origins. CT angiograms. (**A**) Normal posterior cerebral arteries, coronal slice, anterior view. 1. BA; 2. right SCA; 3. right PCA; 4. left PCA; 5. left SCA. (**B**) Fetal type of the right posterior cerebral artery, three-dimensional volume rendering, posterior view. 1. BA; 2. left SCA; 3. left PCA; 4. right fPCA; 5. right SCA.

Type F of the bilateral combination refers to bilateral type 2 and terminal SA. In the overall group, type F was detected in 9.76% (20/205 cases). This type F had the following two subvariants: in 12 cases, the two PCAs originated from the BA (Figure 4A), and in 8/20 subjects with type F, we identified the fetal type of PCA (fPCA). The second subvariant, therefore, appeared morphologically as a trifurcation of AB (Figure 4B).

**Figure 5:**
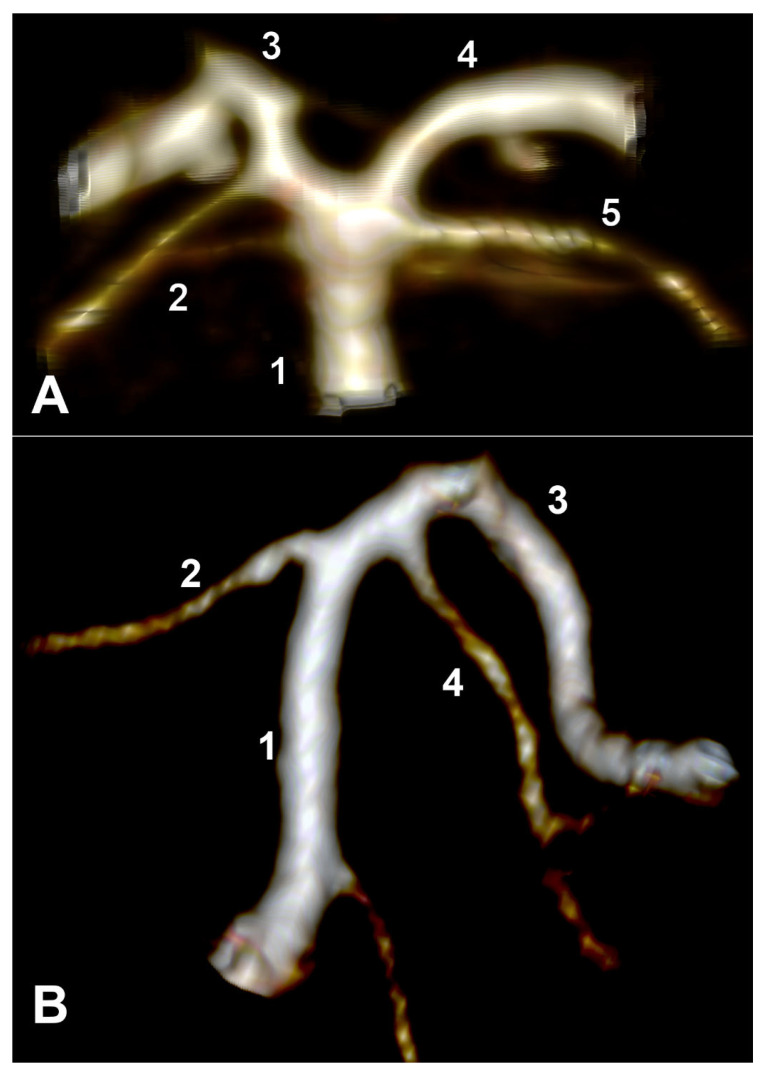
Type G of the bilateral combination of superior cerebellar artery origins. Posterior views of basilar arteries. CT angiograms with three-dimensional volume renderings. (**A**) Bilateral normal PCAs. 1. BA; 2. left SCA; 3. left PCA; 4. right PCA; 5. right SCA. (**B**) Aplastic P1 segment of the left PCA. 1. BA; 2. left SCA; 3. right PCA; 4. right SCA.

In type G of the bilateral combination, one SCA was terminal type 2, and the contralateral one was of cerebral type 3. This bilateral combination also had the following two subvariants: associating bilateral normal PCAs (Figure 5A) with the unilateral absence of the P1 segment of the PCA, resulting in a type 2 SCA, respectively (Figure 5B).

**Figure 6:**
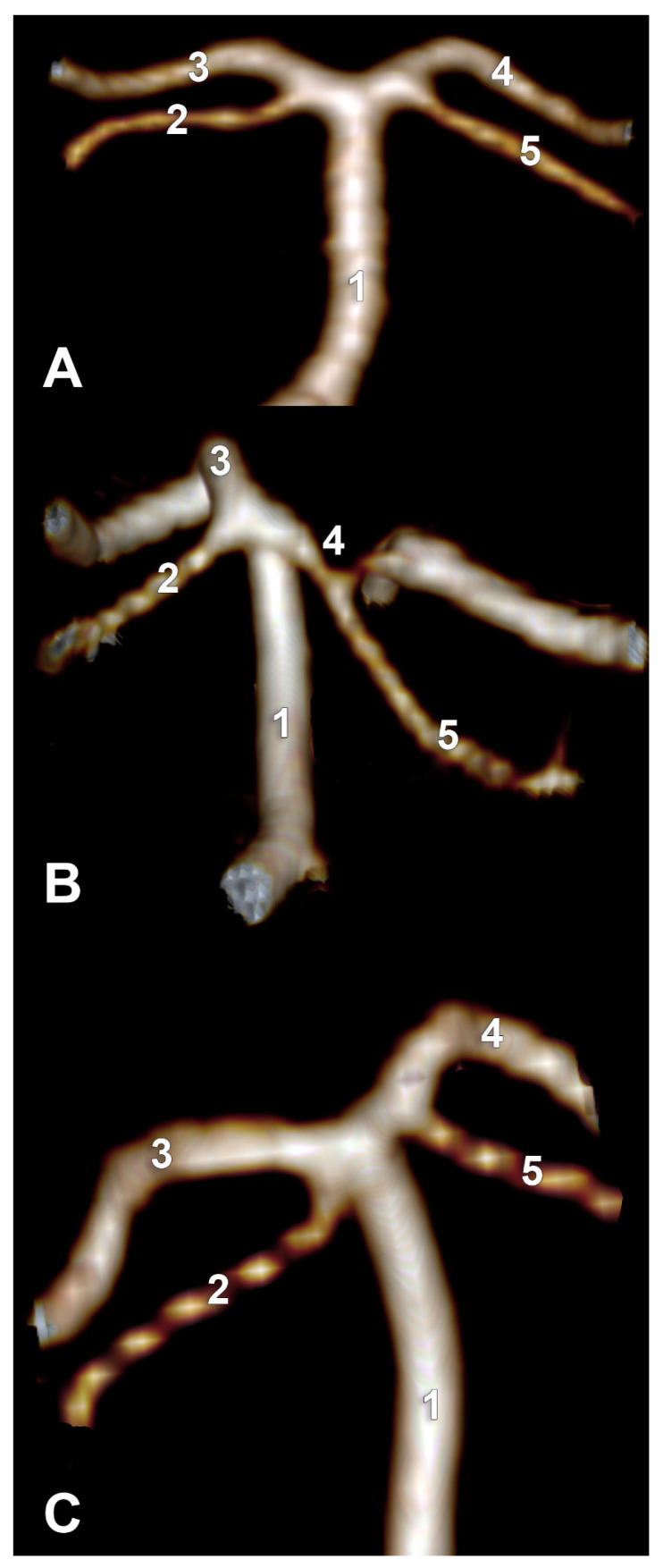
Type H of the bilateral combination of superior cerebellar arteries origins. Posterior views of basilar arteries. CT angiograms with three-dimensional volume renderings. (**A**) Bilaterally symmetrical PCA origins of SCAs. (**B**) The hypoplastic right P1 segment of PCA. (**C**) Bilaterally asymmetrical PCA origins of SCAs. 1. BA; 2. left SCA; 3. P1 segment of left PCA; 4. P1 segment of right PCA; 5. right SCA.

In type H, both SCAs originated from PCAs. The distance between the origins of SCA and PCA was variable. SCA originated either from a normoplastic PCA or from a hypoplastic P1 segment. When SCA and PCA-P1 calibres were comparable but smaller than the P1 segment proximal to the SCA origin, this segment could also be regarded as a common trunk of SCA and P1 (Figure 6).

**Figure 7:**
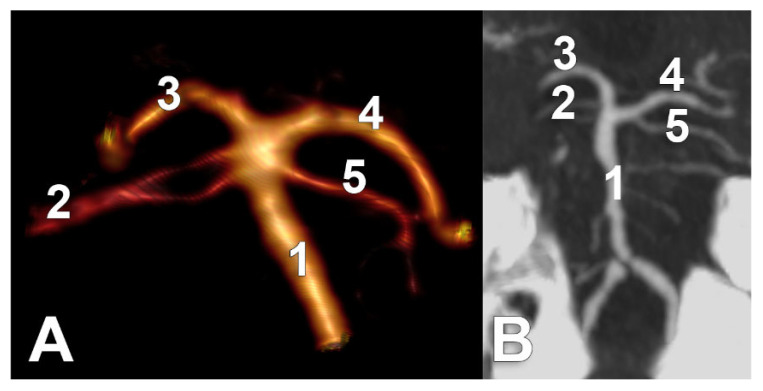
Unilateral duplicated SCAs. (**A**) Type E bilateral combination. Three-dimensional volume rendering, posterior view. 1. BA; 2. duplicated left SCA; 3. left PCA; 4. right PCA; 5. right SCA of collateral type 1. (**B**) Type I of the bilateral combination. Coronal MPR slice through the vertebrobasilar arteries, anteriorly viewed. 1. BA; 2. Right-duplicated SCA; 3. right PCA; 4. left PCA; 5. The left SCA of cerebral type 3.

Unilateral duplicated SCAs were found in types E and I and associated contralaterally with either the collateral type 1 of SCA or the cerebral type 3 of SCA, respectively (Figure 7). There were no bilateral duplications of SCA.

**Figure 8:**
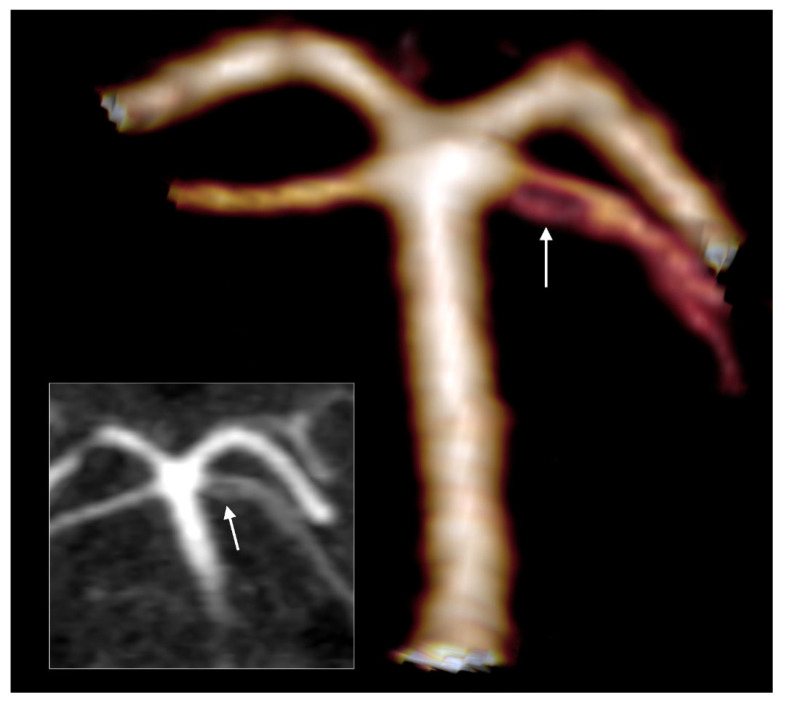
Fenestrated superior cerebellar artery. Three-dimensional volume rendering and coronal MPR (inset). Posterior views. The arrow indicates the fenestration of the right SCA.

Fenestrated SCA was found in a single J-type case and associated with the contralateral SCA of collateral type 1 (Figure 8).

Some bilateral combinations have led to modified BA endings. These bilateral combinations included unilateral type 2 (terminal) with combinations C (types 1 + 2), F (types 2 + 2) and G (types 2 + 3). Considering the cases with fPCA and aP that were present, we defined the following subtypes.

**Figure 9:**
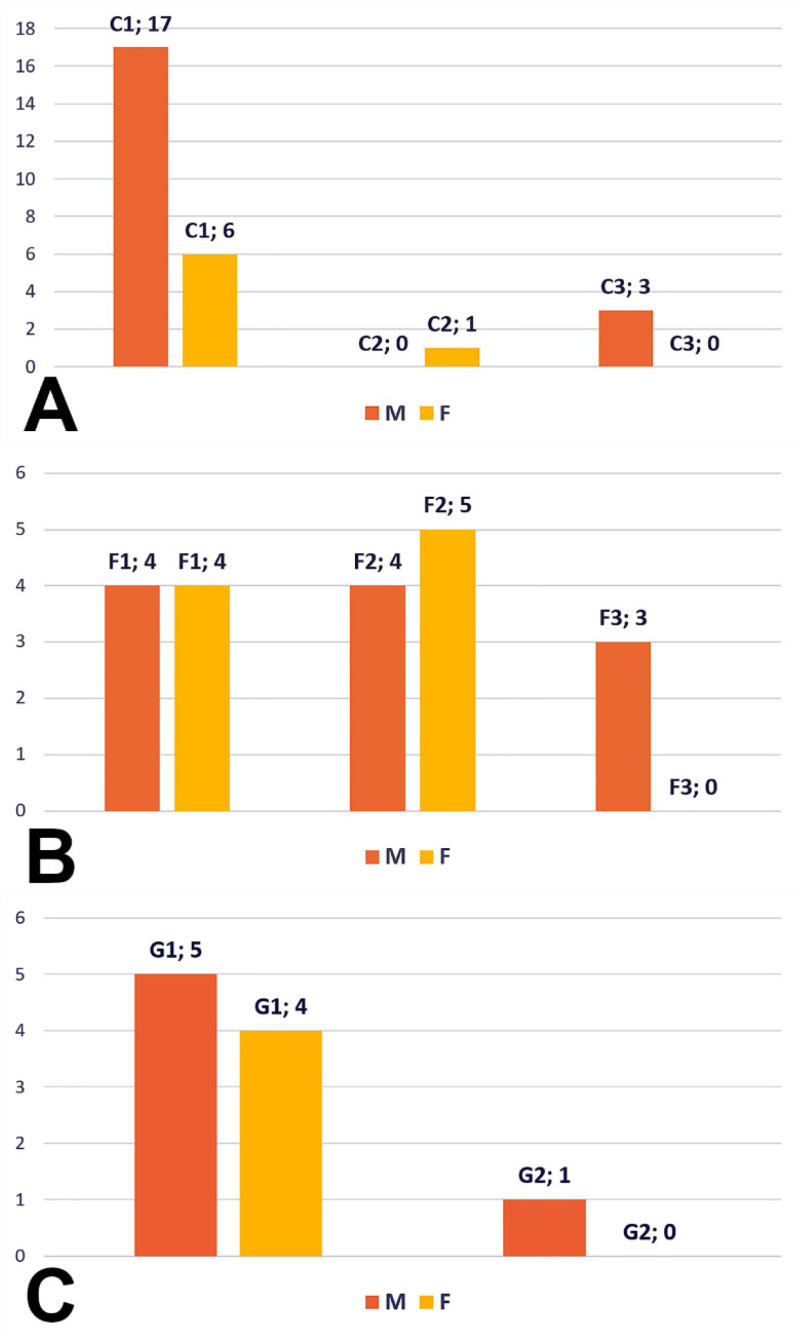
(**A**) Bar chart of the subtypes of the C-type combination (count) of superior cerebellar artery origins (types 1 and 2). M: males; F: females. (**B**) Bar chart of the subtypes of the F-type combination (count) of superior cerebellar artery origins (bilateral type 2). M: males; F: females. (**C**) Bar chart of the subtypes of the G-type combination (count) of superior cerebellar artery origins (type 2 and type 3). M: males; F: females.

Type C (27 cases, 20 male and seven female) resulted in the following: subtype C1—BA trifurcation with bilateral PCA and unilateral SCA of the terminal type, subtype C2—BA trifurcation with fPCA, type 2 SCA, P1 segment of PCA and aP, and subtype C3—BA bifurcation with unilateral fPCA with type 2 SCA on that side and the P1 segment on the side with the SCA of collateral type 2 (Figure 9A).

Type F (20 cases, 11 males, nine females) resulted in the following: subtype F1—quadrifurcated BA, subtype F2—fPCA and trifurcated AB, and subtype F3—bilateral fPCA and BA bifurcated into SCAs (Figure 9B).

**Figure 10:**
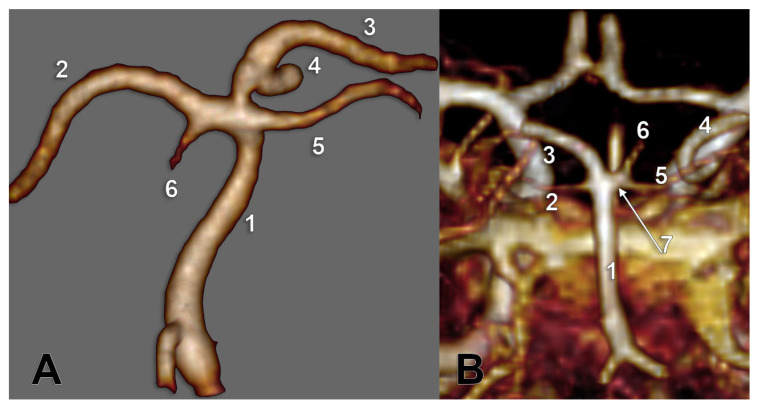
(**A**) Posterior view of the basilar artery, with a computed tomography angiogram, and three-dimensional volume rendering. The right posterior cerebral artery appears as a superior median branch of a trifurcated basilar artery. 1. BA; 2. left PCA; 3. right PCA; 4. posterior communicating artery; 5. right SCA; 6. left SCA. (**B**) Common trunk of origin of the superior cerebellar artery and the artery of Percheron. MR angiogram, three-dimensional rendering. Posterior view. 1. BA; 2. left SCA; 3. left PCA; 4. fPCA; 5. right SCA; 6. aP; 7. common trunk of right SCA and aP.

For type G (10 cases, six males, four females), we observed two subtypes (Figure 9C): G1—BA trifurcation, with the two P1 segments of the PCA and with one terminal type 2 of SCA (Figure 4B) and subtype G2—BA bifurcation, with fPCA and terminal type 2 SCA, but with the standard contralateral P1 segment of PCA giving off a type 3 SCA (Figure 5A). One male case showed a G-type bilateral combination but with a median insertion of the PCA into the basilar end (Figure 10A).

Another male case was found with bilateral combination type D (1 + 3), which can lead to a bifurcated BA terminal morphology with two ACPs. But, on the right side, it showed fPCA, with an absent P1 segment with the terminal basilar branch on that side being a thick common trunk of SCA and aP (Figure 10B).

Also, in the cases with duplicate SCA (combinations E and I), the insertion in the arterial axis of the double SCA caused either bifurcations or the trifurcation of the BA; thus, of the four cases of type E, one had a double SCA with both type 1 collateral trunks and, therefore, AB bifurcation and the other three had each double SCA with one type 1 collateral trunk and one type 2 terminal trunk, and thus BA trifurcation. The case with the type I combination had double SCA with one type 2 terminal trunk and one type 3 cerebral trunk, leading to BA trifurcation.

## 4. Discussion

Krzyzewski et al. (2014) defined the following anatomical types of SCA origin: type 1—both SCA originate from BA (74%); type 2, aplastic SCA (4%), with subtypes 2a, which are bilateral (2%) and 2b, which are unilateral (2%); type 3, duplicated SCA (5.5%) with subtypes 3a, which are bilateral (0.5%) and 3b, which are unilateral (5%); type 4, common origin of SCA and PCA (corresponding to type 2 in the present study) (9.5%) with subtypes 4a, which are bilateral (2%) and 4b, which are unilateral (7.5%); and type 5, SCA from PCA (corresponds to type 3 in the present study) (9%), with subtype 5a bilateral, not identified in the study, and subtype 5b, unilateral, found in 9% of cases [21]. The respective authors indicate the association of subtype 5b with contralateral SCA of the terminal type [21]. The 1 + 3 combination of types in the present study and type D’s bilateral morphological combination had a prevalence of 8.29% in the overall group, including 10.17% in men and 5.75% in women [21].

Pekcevik and Pekcevik (2014) published the results of a study of 341 CT angiograms [5]. The authors distinguish between the “common trunk” of PCA and SCA and the variant of SCA originating from PCA, respectively [5]. The difference, as exemplified in specific figures of that article, is that the common trunk in question is thicker than the continuation of the P1 segment distal to the SCA origin. By contrast, the SCA origin from the PCA corresponds to a relatively uniform calibre of the P1 segment of PCA [5]. This common trunk can be seen either as the initial thicker subsegment of the P1 segment of the PCA or as a normal subsegment of P1 followed by a hypoplastic one distal to the origin of SCA.

A dissection study on 173 specimens identified only two types of SCA origin as follows: the direct origin from the BA, in 93.4% of cases, either as a single artery (72.1%) or with duplicated SCAs (21.3%) [18]. The duplication of SCA was unilateral in 12.3% of cases and bilateral in 2% of cases [18]. The authors describe that the SCA on the left in the common trunk with the PCA (similar classification to Pekcevik and Pekcevik, 2014) in 2.5% of cases; in four of these cases, the SCA was duplicated [18]. The origin of SCA from PCA was found in 4% of cases [18]. The authors present evidence for that so-called common trunk origin but had no picture of the SCA origin from PCA [18], which could have helped with the morphological distinction.

In the present study, bilaterally symmetrical types of SCA origin were found in 70.7% (overall group), 64.4% (male group), and 78.16% (female group). No bilateral SCA duplication was found. In a group of 93 cadavers, the symmetrical origin of SCA occurred in 43%, bilateral asymmetry in 57%, and unilateral duplication in 22.1% [10].

Bala et al. (2013) published a study on the BA trifurcation [22]. The authors report the SCA’s unilateral origin from the BA, which determines the trifurcation [22]. In the present study, we highlighted different BA trifurcation possibilities associated with types C, F, and G of bilateral combinations.

Gunnal et al. (2015) studied how the BA ends in 170 brain preparations [23]. In Table 2, we present the morphological patterns of different authors compared with those resulting from the present study, which appear to be more diverse and detailed. We considered the types described by Gunnal as common trunks of PCA and SCA [23] and as cerebral type 3 of SCA, arising from PCA. Gunnal also shows the so-called trifurcation of the BA resulting from the addition of the posterior communicating artery; this latter, however, appears in the image presented by the authors as inserted in the initial segment of the PCA and not precisely in the BA tip. Gunnal et al. described the pentafurcations of the BA by including double SCAs in the counts and without distinguishing the origins of SCAs as collateral/terminal/cerebral types [23]. Similarly, Nagawa et al. (2018) found penta- and hexafurcations of BA determined by double or triple SCAs, but they did not discriminate SCAs’ exact sites of origin [24].

Gunnal et al. described the situations with an absent P1 segment as non-furcation, making the SCA appear on that side of terminal type 2, with the contralateral P1 segment inserted medially at the apex of AB [23]; this morphology corresponds to the C (1 + 2) and G (2 + 3) types of bilateral combinations in the present study and should be correlated to certain furcations of the BA. In a case presented by Gunnal as non-furcation, an aP was also seen in the image, determining a trifurcated pattern of the basilar tip [23]. The respective authors did not specifically document the presence of FPCAs in their study [23].

Stopford (1916) found the origin of the SCA to be constant; in 94% of a batch of 150 brain specimens, the origin of the SCA was at the upper boundary of BA, virtually at the upper edge of the pons, and immediately caudal of the point where the BA divides into the two PCAs [8]. In 6% of Stopford’s batch, the origin of the SCA was lower, so the relationship of the SCA to the oculomotor nerve was more distant; in only 1 case out of 150 was the origin of SCA distinguished to be from the BA at the junction of the middle 3rd with the upper 3rd of the BA [8]. Stopford did not detail the microanatomy of the SCA origin, as was considered in the present study, in types 0–3. He also did not identify the fenestrations of SCA [8]. Stopford’s batch is more consistent than the batch used by Rhoton Jr.’s group to study the microsurgical anatomy of SCA [25].

According to Bergman’s Encyclopedia of Human Anatomical Variations, the SCA is duplicated in 14% of hemispheres, with the duplicated vessels corresponding to the rostral and caudal trunks of a normal SCA [26]. The absence of SCA is infrequent [25,26]. In a batch of 150 brain specimens, the absence of the SCA was identified only once by Stopford (1916), unilaterally on the left, and replaced by PCA branches [8]. Stopford cites Longo (1905) with a similar case but also indicates that a study of 220 specimens did not identify the absence of SCA [8]. Hardy et al. (1980) found no absent SCA in a batch of 50 SCAs investigated [25]. In 7/50 specimens, however, Hardy et al. found duplications at the origin of SCA [25]. Stopford (1916) also identified in 150 brain specimen duplications of the SCA: in 12% on the right side, in 16% on the left side, and 3% in bilateral duplications [8]. In one case, he found the triplication of SCA [8]. Stopford also documented Blackburn’s (1907) results as follows: right-sided SCA duplication in 2%, left-sided in 1%, and bilateral in 1% of cases [8]. Pekcevik and Pekcevik (2014) found duplicated SCAs in 29.5% of 314 CT angiograms [5]. In the present study, no bilateral SCA duplication was found; the prevalence of right SCA duplication was only 0.48%, and on the left, it was 1.95%. Although the group used in the present study is more significant than that of Stopford (1916), the prevalence of SCA duplication is consistently lower than his results but correlates with Blackburn’s prevalence. SCA duplication may result from the origin of SCA’s lateral branch being directly from the BA [27].

Uchino (2003) reported an extremely tortuous SCA [28]. Such a rare congenital variant of SCA has also been found on an MRI examination [11]. This variant was not found in the present study.

Thrombotic and embolic occlusions of the SCA cause effects in the spectrum from silent occlusion to the upper brainstem or cerebellar infarction [10]. It has been estimated that of all strokes, cerebellar infarction accounts for 1.5–4.2% in autopsy series and 0.6–1.1% in CT series [29]. Cerebellar infarcts in the SCA territory may occur due to spontaneous vertebrobasilar dissection and are rare in children and adolescents [30]. Infarction in the SCA territory presents a broad spectrum of clinical manifestations, such as rostral basilar artery syndrome, deep coma with quadriplegia, cerebellovestibular syndrome, and various SCA syndromes [27].

Aneurysms of SCA are unusual and rare (0.2–1.7%) [31,32,33,34]. They usually occur at the junction of the BA and SCA (laterobasilar aneurysm) and distal to the SCA (hemispheric aneurysm); proximal SCA aneurysms are more common than distal ones [34]. Bilateral aneurysms at the origin of SCA are sporadic [34]. Because the proximal segment of the SCA has close connections to cranial nerves 3, 4, and 5, SCA aneurysms can cause the paralysis of these nerves [31]. Aneurysms forming from the distal portion of the SCA are sporadic, accounting for less than 0.2% of all cases of intracranial aneurysms [35]. Aneurysms of SCA may be associated with infratentorial arteriovenous malformations [36].

In 29 patients with SCA aneurysms, four anatomical possibilities were found for the location: superior lateral (69%) at the origin of a type 2 SCA (according to the authors’ diagram), horizontal lateral (20.7%), and inferior lateral (6.9%) at the origin of a type 1 SCA, and posterior (3.4%) to the basilar origin of SCA [32]. One can thus reasonably speculate that the lateral location of SCA aneurysms could be favored by the morphological pattern in type 1 SCA, as defined in the present study. However, the subtypes of SCA aneurysms do not differ significantly in treatment modality [32].

Although the surgical treatment of SCA aneurysms is associated with good results in experienced hands, surgical access can be difficult, requiring destructive approaches [31]. Major perforating arteries and adjacent cranial nerves make clipping SCA aneurysms difficult; complications may occur [31]. Because SCA aneurysms are rare, not many surgeons gain experience in treating these aneurysms [31].

Basilar tip aneurysms are complex lesions for microneurosurgical and endovascular therapy [37]. In patients where direct clipping and coil embolization are not options, cerebral revascularization and BA occlusion are possible treatment strategies [37]. Knowledge of vascular anatomical details at the level of the bifurcation of the BA proves its great usefulness in the surgery of BA and SCA aneurysms. Although basilar bifurcation aneurysms are more common, SCA aneurysms are more favourable for microsurgical clipping because they are not intimate with thalamoperforating arteries but take into consideration the different variational possibilities of the SCA; the aneurysm origin must be well documented and understood from preoperative radiological studies (cerebral CT—angiogram or cerebral digital subtraction angiography). The SCA aneurysm pushes away the thalamoperforators along the posterior-superior walls of the P1 segment and the posterior wall of the basilar artery. They lie at the midline, below the basilar bifurcation, and well within the carotid-oculomotor triangle, which improves their visualization relative to basilar bifurcation aneurysms [38].

A good understanding of the local anatomy of the BA bifurcation plays an essential role in clipping basilar bifurcation aneurysms. Identifying the five major arteries (the basilar trunk, the bilateral PCAs, and the bilateral SCAs) gives the neurosurgeon vascular control and anatomic orientation. Knowledge of the origin variants of SCA is essential for the neurosurgeon when dissecting the BA aneurysm neck and placing the clip to occlude it. It is critical to know the position of the contralateral P1 segment or the contralateral proximal SCA because exposure at the depth of the surgical corridor is so limited [39]. The clip blades must be navigated around and under the ipsilateral PCA, SCA, and perforators, with particular attention not to mistakenly occlude the contralateral PCA and SCA. Deep operative corridors, surgical blind spots, and variants of SCA and PCA make the post-clipping inspection especially critical with BA aneurysms.

The surgery of tumours of the petro-clival region (meningiomas, chordomas) that imply complex surgical approaches, such as petrous approaches (Kawase), require the identification and dissection of the arteries at the bifurcation of the BA, the PCA, and the SCA. The importance of knowing the arterial variants of the SCA is given by the fact that arteries that are adherent to the tumour must be dissected and preserved and should be differentiated from those that irrigate the tumour and have to be coagulated. Ischemic lesions in this ponto-mesencephalic region are followed by significant morbidity and mortality.

## 5. Conclusions

The SCA has more anatomical possibilities of origin than presented in anatomical textbooks. There are various bilateral morphological combinations. Therefore, details on the specific end of the BA should be gathered on a case-by-case basis.

## Figures and Tables

**Table 1:** Bilateral combinations of superior cerebellar artery origin types. DUP—duplication. FEN—fenestration.

Type of Bilateral Combination	Unilateral Types
A	1, 0
B	1, 1
C	1, 2
D	1, 3
E	1, DUP
F	2, 2
G	2, 3
H	3, 3
I	3, DUP
J	1, FEN

**Table 2:** Morphological patterns of the basilar artery furcation are consistent with other studies [23,24] and the present study. BA: basilar artery; PComA: posterior communicating artery; PCA: posterior cerebral artery; fPCA: fetal type of posterior cerebral artery; SCA: superior cerebellar artery; aP: artery of Percheron.

Furcation Pattern of the BA	Gunnal et al. (2015)	Nagawa et al. (2018)	Present Study
bifurcation	PCA + PCA (normal)	PCA + PCA (normal)	PCA + PCA (normal) (C3) PCA with ipsilateral type 1 SCA + type 2 SCA with ipsilateral fPCA (F3) SCA + SCA (bilateral fPCA) (G2) PCA with ipsilateral type 3 SCA + type 2 SCA with ipsilateral fPCA PCA + common trunk of SCA and aP
trifurcation	PCA + PCA + SCA PCA + PCA + PComA (?)	PCA + PCA + SCA	(C1) PCA with ipsilateral type 1 SCA + PCA with ipsilateral type 2 SCA (C2) PCA with ipsilateral type 1 SCA + type 2 SCA with ipsilateral cu fPCA + aP (F2) PCA with ipsilateral type 2 SCA + type 2 SCA with ipsilateral fPCA (G1) PCA with ipsilateral type 3 SCA + PCA with ipsilateral type 2 SCA
quadrifurcation	2 PCA + 2 SCA	2 PCA + 2 SCA	(F1) 2 PCA + 2SCA

## Data Availability

No new data were created or analyzed in this study. Data sharing does not apply to this article.
